# Macrophage M1 polarization mediated via the IL-6/STAT3 pathway contributes to apical periodontitis induced by *Porphyromonas gingivalis*

**DOI:** 10.1590/1678-7757-2022-0316

**Published:** 2022-11-21

**Authors:** Xuan Chen, Jinge Dou, Zhuohui Fu, Yang Qiu, Ling Zou, Dingming Huang, Xuelian Tan

**Affiliations:** 1 Sichuan University Department of Operative Dentistry and Endodontics West China Hospital of Stomatology Chengdu China National Clinical Research Center for Oral Diseases & State Key Laboratory of Oral Diseases, West China Hospital of Stomatology, Department of Operative Dentistry and Endodontics, Sichuan University, Chengdu, China.

**Keywords:** IL-6/STAT3, Macrophage, *Porphyromonas gingivalis*, Apical periodontitis, Bone lesion

## Abstract

**Objective:**

To investigate the involvement of IL-6/STAT3 signaling pathway activation in macrophage polarization and bone destruction related to apical periodontitis (AP) stimulated by *Porphyromonas gingivalis*.

**Methodology:**

Macrophage polarization, IL-6/STAT3 expression, and the presence of *P. gingivalis* were detected in human AP tissues via RT-qPCR, western blotting, and immunohistochemistry staining. Murine bone marrow derived macrophages were isolated and cultured with *P. gingivalis* W83 *in vitro*, and levels of macrophage IL-6 expression, STAT3 phosphorylation, and macrophage polarization with or without the selective STAT3 phosphorylation inhibitor Stattic (5 μM) were detected via ELISA, western blotting, RT-qPCR, and flow cytometry, respectively. *P. gingivalis*-induced murine AP models were constructed, and bone destruction and macrophage polarization in the apical region were evaluated. Transwell co-culture systems were used to investigate the effects of macrophages infected with *P. gingivalis* on osteogenesis and osteoclastogenesis.

**Results:**

*P. gingivalis* was detected in human AP tissues that highly expressed IL-6/STAT3, and the M1 subtype of macrophages was more abundant in these tissues. *P. gingivalis* infection induced IL-6 expression, STAT3 phosphorylation, and M1 polarization of macrophages, while 5 μM of Stattic partially abolished these activation effects. Systemic STAT3 blockade via oral administration of Stattic at a dose of 25 mg kg-1 alleviated murine periapical bone resorption and apical infiltration of M1 macrophages induced by *P. gingivalis* infection *in vivo*. Furthermore, macrophages infected with *P. gingivalis* promoted bone destruction via secretion of IL-6, TNF-α, and RANKL, which hinder pre-osteoblast expression of Runx2 and accelerate pre-osteoclast expression of NFAT2.

**Conclusions:**

The activation of IL-6/STAT3 signaling pathway is involved in mediating macrophages M1 polarization in the *P. gingivalis* induced apical inflammatory context and may also be intimately involved in the bone loss caused by *P. gingivalis* infection, directing the M1 macrophage infiltration during the progression of AP.

## Introduction

Apical periodontitis (AP) is an infectious inflammatory lytic bone lesion, primarily caused by microbial infection of the root canal system.^1,2^ Invasion of bacteria and their components elicits a nonspecific host response in periapical tissues that results in local inflammation, infiltration of immune cells, resorption of hard tissues, formation of granulation tissue, and eventual development of various periapical lesions.^3,4^

*Porphyromonas gingivalis*, the so-called keystone pathogen in periodontitis,^5^ is detected not only in the biofilm in periodontal pockets, but also as a major bacterial species in infected pulp chambers and root canals with AP.^3,6,7^ However, the mechanism underlying the involvement of *P. gingivalis* in the pathology of AP has yet to be elucidated. *P. gingivalis* uses several strategies to interact with the host defense system, escape host immune surveillance, and initiate the inflammation process; this bacterial pathogen can invade and survive inside endothelial cells, osteoblasts, and macrophages.^8–10^ By activating Toll-like receptors (TLRs), *P. gingivalis* induces the expression of cytokines including interleukin-6 (IL-6), granulocyte colony-stimulating factor (G-CSF), and lymphotactin in murine macrophages,^11,12^ causing murine bone loss.^13–15^

Macrophages are indispensable immunocompetent infiltrates in the development of AP.^16^ M1 and M2 polarized macrophages are two major types of activated macrophages.^17^ Classically activated M1 macrophages produce pro-inflammatory and bone-destructive mediators. such as IL-1α, IL-6, IL-1β, and tumor necrotic factor-α (TNF-α), which lead to cytotoxicity, tissue injury, and excessive osteoclastic activity. In contrast, alternatively activated M2 macrophages secrete anti-inflammatory mediators, such as IL-10 and arginase 1, which regulate the level of chronic inflammation and are further associated with tissue homeostasis, resolution of inflammation, and tissue repair.^16,18–20^ Recently, however, the immunomodulatory state of M2 macrophages has been found to be related to maintenance of periapical granuloma.^21^ Balance between M1 and M2 is crucial in osteoblast differentiation derived from mesenchymal stem cell, which also affects the decay of bone homeostasis and regeneration during chronic inflammation.^19,22^ Therefore, macrophages, especially polarized macrophages and macrophage-derived cytokines, are potential targets for the immunomodulation and improvement of AP.^16,21^ However, the conditions underlying macrophage polarization in AP tissues remain controversial.^4,23,24^

Pro-inflammatory cytokine IL-6 is instrumental in chronic inflammatory diseases and autoimmune diseases, as well as bone metabolism, and predominantly exerts its effect through the IL-6-signal transducer and activator of transcription 3 (STAT3) pathway. IL-6 transcription is induced by various stimuli, such as TLR ligands, IL-1, and TNF-α.^25^ *P. gingivalis* infection can disrupt oral biofilm balance and induce host responses that lead to overexpression of IL-6 and subsequent destruction of periodontal tissues.^22,26^ Studies in oral diseases have speculated that IL-6 is an important marker for periodontitis.^27,28^ Blocking the IL-6/STAT3 signaling pathway using the novel nonpeptidic inhibitor Stattic demonstrated promising potential in counteracting osteoclast differentiation and bone resorption in osteoarthritis.^29^ However, the involvement of the IL-6/STAT3 pathway in the initiation and progression of AP, another oral lytic bone disease, has yet to be explored. Activation of the IL-6/STAT3 pathway is also reported to drive macrophages toward M2 differentiation in the tumor microenvironment.^25^ Nevertheless, as a chemokine highly expressed by M1 macrophages, the effects of IL-6 and the related IL-6/STAT3 pathway on macrophage polarization in the AP context remains to be elucidated.

Our study aims to explore if the IL-6/STAT3 signaling pathway was involved in *P. gingivalis*-induced macrophage M1 polarization, and if the M1-polarized macrophages would contribute to the progression of AP and bone destruction.

## Methodology

### Ethics statement

All animal experimental procedures were conducted in strict accordance with the ethical protocol permitted by the Ethics Committee of West China School of Stomatology, Chengdu, China (approval number: WCHSIRB-D-2020-041), and the methods were carried out in accordance with the approved guidelines. Clinical sample collection and laboratory examination of the tissue samples were also conducted in strict accordance with the ethical protocol permitted by the Ethics Committee of West China Hospital of Stomatology, Chengdu, China (approval number: WCHSIRB-D-2020-056).

### Clinical samples collection

The human AP group comprised six patients (three males and three females) that were referred to the Department of Conservative Dentistry and Endodontics between April 2020 and December 2020. Patients ranged in age from 20 to 60 years (mean 36.3 years), and none of the patients reported a history of systemic disease. All patients had a history of non-surgical initial root canal treatment performed over one year prior to the study and were diagnosed according to radiographic and clinical examinations with persistent apical periodontitis in previously treated teeth. All affected teeth had intact coronal restorations and a probing depth of less than 4 mm, with physical mobility and no signs of root fracture. All patients elected to treat the affected teeth via endodontic microsurgery and signed a consent statement.

Periapical lesion samples were collected during the endodontic microsurgeries. Prior to the intervention, the patients were instructed to rinse their mouths with 2% povidone iodine solution to reduce bacterial load inside the oral cavity. After regular local anesthesia, flap reflection, and osteotomy, the inflamed tissues surrounding the affected root ends were carefully curetted, swiftly rinsed with sterile saline, and had a section removed with sterile scissors. Parts of the tissues removed were snap-frozen in liquid nitrogen, preserved at −80°C, and used for subsequent molecular biological examinations. The remaining tissue parts were fixed in formalin, dehydrated, and embedded in paraffin for immunofluorescence staining. When excising the periapical inflamed tissues, special care was taken to protect the samples from saliva and gingival fluid contamination.

Healthy dental pulp tissues collected from healthy, intact third molars were used as negative controls. Six patients with no systemic disease history or periodontitis were enrolled in this healthy group (male, *n*=2; female, *n*=4; mean age, 22±1.5 years), and written consent was also obtained.

### Tissue DNA extraction

Genomic DNA of the frozen samples from the human AP and healthy groups were enzymatically extracted with the QIAamp DNA Mini Kit (Qiagen, Dusseldorf, Germany), following the manufacturer's instructions. The yielded gDNAs were used to determine the presence of *P. gingivalis* in the tissues via real-time quantitative polymerase chain reaction (RT-qPCR) with *P. gingivalis*-specific primers, as described below.

### Immunofluorescence staining based on the tyramide signal amplification (TSA) method

Paraffin-embedded human AP lesion tissues were cut into serial slices with a thickness of 4 μM. After routine deparaffinization and dehydration, immunofluorescence staining was performed with the TSAPlus Triple Fluorescence Staining Kit (Servicebio, Wuhan, China). According to the manufacturer's recommendation, the slides containing the tissue slices were successively incubated with primary antibodies against CD206 (1:1000, Abcam, #ab64693, Cambridge, MA, USA), iNOS (1:1000, Huabio, #ER1706-89, Hangzhou, China) and CD68 (1:1000, Invitrogen, #14-0688-82, Carlsbad, CA, USA). Phosphate-buffered saline (PBS) was used as a negative control. The stained slices were digitally scanned and recorded using the 3DHISTECH biopsy scanner (3DHISTECH, Budapest, Hungary).

### Isolation and culture of bone marrow-derived macrophages (BMDM)

Specific pathogen-free (SPF) male C57BL/6J mice (Dashuo Experimental Animals Co. Ltd, Chengdu, China), aged 7-8 weeks, were adopted for BMDM generation. Murine femurs were aseptically harvested, and the contents of the femurs were flushed out with Roswell Park Memorial Institute 1640 (RPMI1640) medium (Gibco, Thermo Fisher, NY, USA), supplemented with 5% fetal bovine serum (FBS) (Gibco) and 1% 100 U mL^-1^ penicillin G and 100 μg mL^-1^ streptomycin (Sigma Aldrich, St Louis, MO, USA). Bone marrow cells were seeded at 1×10^6^ cells mL^-1^ in complete RPMI1640 (RPMI1640 supplemented with 20% (vol/vol) FBS, 1% 100 U mL^-1^ penicillin G and 100 μg mL^-1^ streptomycin), 20 μM β-mercaptoethanol (Sigma Aldrich), and 10 ng mL^-1^ macrophage colony-stimulating factor (M-CSF) (Peprotech, Rocky Hill, NJ, USA). Cells were seeded in 10-cm cell culture petri dishes (Falcon, Corning, NY, USA) and grown for seven days to generate mature BMDM (designated as BMDMφ), which could be subjected to subsequent stimulation and analysis.

### Bacterial culture and macrophage infection

*P. gingivalis* W83 was grown in brain heart infusion broth (BHI) (Oxiod, Basingstoke, UK) enriched with hemin (5 g mL^-1^) (Solarbio, Beijing, China) and menadione (1 g mL^-1^) (Solarbio), in an anaerobic atmosphere (85% N_2_, 10% H_2_, 5% CO_2_), at 37°C. Bacteria were collected when the culture reached an optical density at 600 nm of approximately 1.0 (~10^9^ CFU mL^-1^) and were used for infection of unprimed BMDMφ at a multiplicity of infection (MOI) of 100, ^30^ in the presence or absence of 5 μM Stattic (Selleck, TX, USA), a nonpeptidic small molecule that selectively inhibits the phosphorylation of STAT3. *P. gingivalis* W83 were incubated with the unprimed BMDMφ for 12 h.

### Transwell co-culture

BMDMφ infected with *P. gingivalis* W83 (MOI=1:100) for 12 h, with or without Stattic, were harvested, seeded in the upper chamber of a 6-well Transwell plate (size 0.4 μm, Corning, NY, USA) (2 × 10^5^ cells mL^-1^), and cultured with RPMI1640 supplemented with 20% (vol./vol.) FBS, 1% 100 U mL^-1^ penicillin G, and 100 μg mL^-1^ streptomycin (designated as M0 culture medium). For analysis of osteogenic effects, MC3T3-E1 pre-osteoblasts were seeded (2 × 10^5^ cells mL^-1^) in the bottom chamber of the Transwell plate, in osteoblastic-induced medium (obi) comprising α-Minimum Essential Medium (α-MEM) (Procell, Wuhan, China) supplemented with 10% (v/v) FBS, 10 mM β-glycerophosphate, 100 nM dexamethasone, 50 ug mL^-1^ ascorbic acid, 100 U mL^-1^ penicillin G, and 100 μg mL^-1^ streptomycin. For analysis of osteoclastic effects, RAW264.7 pre-osteoclasts were seeded (2 × 10^5^ cells mL^-1^) in the bottom chamber in complete high-glucose Dulbecco's Modified Eagle Medium (hi-glu DMEM) (Procell). Acellular upper chambers containing only M0 culture medium were paired with either MC3T3-E1 cells cultured in obi or RAW264.7 cells cultured with hi-glu DMEM supplemented with 50 ng mL^-1^ Receptor Activator for Nuclear Factor-κ B Ligand (RANKL) (Peprotech) in the bottom chambers, establishing the positive controls for osteogenic and osteoclastic inductions, respectively. The Transwell plates were incubated in a humidified atmosphere of 5% CO_2_ at 37°C for 3 days, then total proteins of cells in the bottom chambers were collected for western blot analysis.

### Enzyme-linked immunosorbent assay (ELISA)

Concentrations of secreted IL-6, TNF-α, and RANKL in the culture supernatant of *P. gingivalis*-infected BMDMs were measured using commercially available ELISA kits (mmbio, #14205 for IL-6, #919 for TNF-α, and #1552 for RANKL, Yancheng, China), according to the manufacturer's instructions. The detectable ranges of IL-6, TNF-α, and RANKL concentrations were 0-120 pg mL^-1^, 0-640 pg mL^-1^, and 0-200 pg mL^-1^, respectively.

### Flow cytometry (FC) analysis

BMDMs stimulated with 1 μg mL^-1^ *P. gingivalis* lipopolysaccharide (LPS) (InvivoGen, San Diego, CA, USA) plus 20 ng mL^-1^ interferon-γ (IFN-γ) (Gibco) or 20 ng ^-1^ IL-4 (Gibco) were used as M1 and M2 positive controls, respectively, for gating in flow cytometry. Cells infected with *P. gingivalis* with or without Stattic, the positive control groups, and BMDMφ were collected, treated with anti-mouse CD16/32 (TruStain FcX™ PLUS, Biolegend, #156603, San Diego, CA, USA), and then incubated with anti-mouse CD86-fluorescein isothiocyanate (FITC) antibody (clone GL-1, Biolegend, #105005) and anti-mouse CD206-allophycocyanin (APC) antibody (clone C068C2, Biolegend, #141707). according to the manufacturer's instructions. The labeled cells were then analyzed on a Beckman Coulter FC500 flow cytometer (Beckman Coulter Pty Ltd., NSW, Australia), and data were analyzed using FlowJo (v10, BD, Ashland, USA).

### RNA extraction and reverse transcription

TRIzol reagent (Invitrogen) was applied to extract total RNA from the frozen clinical human samples and cultured BMDMs following the manufacturer's instructions. The extracted RNAs were used as reverse-transcribed templates for cDNA synthesis via the PrimeScript RT Reagent Kit (TaKaRa, Shiga, Japan).

### RT-qPCR

The generated gDNAs and cDNAs were subjected to RT-qPCR analysis performed on the Applied Biosystems QuantStudio 7900 machine. The relative expression of *IL-6* and the presence of *P. gingivalis* in the clinical human samples were normalized to internal control β-ACTIN, and comparisons between the healthy and AP groups were made via the reflected 2^-ΔCT^ value.^31^ Expression levels of *IL-6*, *STAT3*, *TNF-*α, and *IL-1β* in BMDMs were normalized to internal control β-actin and reflected in the 2^-ΔΔCT^ values. Primers for human *IL-6* and β-ACTIN, universal primers for *P. gingivali*s, and primers for mouse *IL-6*, *STAT3*, *TNF-*α, *IL-1β*, and β-actin were synthesized by Tsingke Biotechnology Co., Ltd. (Chengdu, China). Table 1 shows the sequences.

**Table 1:** Primers used in this study

	Forward (5′-3′)	Reverse (5′-3′)	References
IL-6 (human)	GAACTCCTTCTCCACAAGCG	ATCTTCTCCTGGGGGTACTGG	68
β-ACTIN (human)	TGACGTGGACATCCGCAAAG	CTGGAAGGTGGACAGCGAGG	68
P. gingivalis	AGGCAGCTTGCCATACTGCG	ACTGTTAGCAACTACCGATGT	69
IL-6	AGTTGCCTTCTTGGGACTG	CCTCCGACTTGTGAAGTGGT	70
Stat3	CCCCCGTACCTGAAGACCAAG	TCCTCACATGGGGGAGGTAG	71
Tnf-α	CCCTCACACTCAGATCATCTTCT	GCTACGACGTGGGCTACAG	72
IL-1β	GCCCATCCTCTGTGACTCAT	AGGCCACAGGTATTTTGTCG	72
β-actin	GACGGCCAAGTCATCACTATTG	CCACAGGATTCCATACCCAAGA	73

### Western blot

Tissue proteins of the frozen clinical human samples and total cellular proteins of the BMDMs, MC3T3-E1 cells, and RAW264.7 cells were extracted with a total protein extraction reagent kit (KeyGEN, Nanjing, China), following the manufacturer's protocol. Exactly 30 μg protein was loaded per well, subjected to 10% SDS-PAGE, and then transferred to PVDF membranes (Millipore, Billerica, MA, USA). The membranes were incubated with non-fat milk for 1 h, then with primary antibodies, including anti-p-STAT3 (1:1000, HuaBio, #ET1603-40), anti-STAT3 (1:1000, HuaBio, #ET1607-38), anti-β-actin (1:1000, Cell Signaling Technology, #4970, Danvers, MA, USA), anti-Runx2 (1:1000, Abcam, #ab264077), anti-Osterix (1:1000, Abcam, #ab22552), and anti-NFAT2 (1:1000, Abcam, #ab25916), overnight at 4°C, followed by incubation with goat anti-rabbit IgG HRP-conjugated secondary antibody (ZSGB-BIO, Beijing, China) for 1 h at room temperature. Relevant protein/antibody bands were detected with an enhanced chemiluminescence detection kit (Epizyme, Shanghai, China) and exposed in a Molecular Imager (Bio-Rad, Hercules, CA, USA).

### Construction of a *P. gingivalis*-derived murine AP model

*P. gingivalis*-derived AP was induced in mice according to methods described in Furusho's work.^32^ The sample size was determined using G*power (version 3.1.9.7) (https://stats.idre.ucla.edu/other/gpower/, UCLA, USA).^33,34^ The sample size was estimated at 12 mice per group in order to achieve a *P* value less than 0.05 with an actual power of over 80%. A total of 48 8-week-old SPF male C57BL/6J mice (Dashuo Experimental Animals Co. Ltd, Chengdu, China) were included in the study. The mice were kept in a SPF environment with a 12 h light/dark cycle and given free access to food and water.

Mice were randomly assigned to different treatments (n=12 per group): no-treatment control (Con group); apical periodontitis (AP group); Stattic treatment (Stattic group); or 0.5% carboxymethylcellulose (Selleck) vehicle treatment (CMC group). AP was induced in all but the Con group. Briefly, after general anesthesia was induced by intraperitoneal injection of 0.4% sodium pentobarbital (J&K Scientific, Beijing, China), the operating area were disinfected with 75% ethanol containing moist sterile cotton swabs, special care was taken to prevent aspiration. Disinfectant was air-dried, then the pulp chamber of the mandibular first molar was exposed using a sterile #1/2 round drill to a depth equal to the diameter of the drill to prevent furcal perforation, with the process performed with a head mount magnifier loupe (Zeiss Inc, Thornwood, NY, USA). Next, a small sterile cotton swab, fully soaked with concentrated *P. gingivalis* W83 PBS suspension at a concentration of 1×10^9^ CFU mL^-1^, was applied in direct contact with the dental pulp for 5 min. Perforation of the pulp chamber was then restored with composite resin (3M ESPE, St. Paul, MN, USA) to induce AP that is derived from *P. gingivalis* infection. All the aforementioned operations inside the mouth were completed with a head mount magnifier loupe (Zeiss Inc, Thornwood, NY). For medication treatment, Stattic (25 mg kg^-1^) or an equivalent volume of vehicle (0.5% carboxymethylcellulose) was administered by oral gavage^35^ every other day from the day after infection induction for a duration of 21 days.

### Micro-computed tomography (Micro-CT) scan and analysis

Mice were sacrificed on day 21 after model construction. The mandibles were removed and fixed with 4% paraformaldehyde for 24 h at 4°C, then transferred into suitable containers filled with 0.5% paraformaldehyde for micro-CT scanning. To evaluate the degree of apical bone destruction around the roots of the first molar, the mandibles were scanned using micro-CT scanner μCT-50 (SCANCO Medical, Bruttisellen, Switzerland). The scanning was performed at 70 kV and 114 mA, with a voxel size of 12 μm, 300 ms integration time, and a 1024 reconstruction matrix. After the scanning, the mandibular first molar and all the surrounding connective tissues were manually selected as the region of interest (ROI) for three-dimensional reconstruction. For apical structural morphometry analysis, the apical ends of the mandibular first molar were identified in the horizontal view of each sample; a shape was drawn on each sample to include all the apical ends of the mandibular first molar, the areas within the shape – starting from the 15^th^ slice coronal to the apical end to bottom of the apical translucent region – were selected as ROI. The three-dimensional reconstruction and analysis of the ratio of bone volume over tissue volume (BV/TV) were carried out with SCANCO VISUALIZATION and SCANCO EVALUATION software (SCANCO Medical), respectively. All measurements were performed by the same researcher, who was blinded to the intervention.

### Hematoxylin and eosin (H&E) and immunohistochemical (IHC) staining

After micro-CT scanning, mandibles of the mice were decalcified with 10% EDTA for 2 weeks, routinely dehydrated, and embedded in paraffin. In parallel to the long axis of the mandibular first molar, the embedded samples were cut into slices of 4 μm thickness. H&E staining was performed using the H&E staining kit (Solarbio) to appraise the degrees of inflammatory infiltration and bone destruction in the periapical area of the mandibular first molars. To analyze macrophage infiltration and polarization states in the murine periapical lesions, IHC staining was performed with primary antibodies including anti-F4/80 (1:500, Invitrogen, #14-4801-81), anti-CD206 (1:100, Abcam, #ab64693), and anti-iNOS (1:100, Huabio, #ER1706-89). Expression of the target molecules was shown by horseradish peroxidase-diaminobenzidine (HRP-DAB) immunostaining (ZSGB-BIO). The stained slices were digitally scanned and recorded using the 3DHISTECH biopsy scanner.

### Statistical analysis

Experimental data were representative of three independent experiments with at least three samples. Mean fluorescence intensity (MFI) and IHC staining results were visualized using the software Caseviewer (https://www.3dhistech.com/solutions/caseviewer/). For the TSA staining slices, 3 independent fields (at the magnification of × 600) from each sample were randomly selected and analyzed for MFI with ImageJ (https://imagej.net/software/fiji/downloads). Upon analysis, only cells presenting double staining for CD68 and iNOS (M1) or CD68 and CD206 (M2) were accounted to avoid the quantification of non-macrophages cells expressing iNOS or CD206. For the IHC staining samples, immunopositive cells in 5 randomly selected fields of the periapical tissue (the tissue around the lower one-third part of the roots of the mandibular first molar) were counted under × 400 magnification. The mean average was estimated from these observations. After confirmation of variance homogeneity of the data, statistical results from two groups were compared using an unpaired *t*-test, and the comparisons between multiple groups were analyzed via one-way analysis of variance (ANOVA) followed by Tukey's test, with SPSS software (version 22.0, IBM, Armonk, NY, USA). *P* values less than 0.05 were considered statistically significant. Results are presented as mean ± SD and marked with “*” for *p*<0.05.

## Results

### M1 macrophages were more abundant in human AP tissues

**Figure 1:**
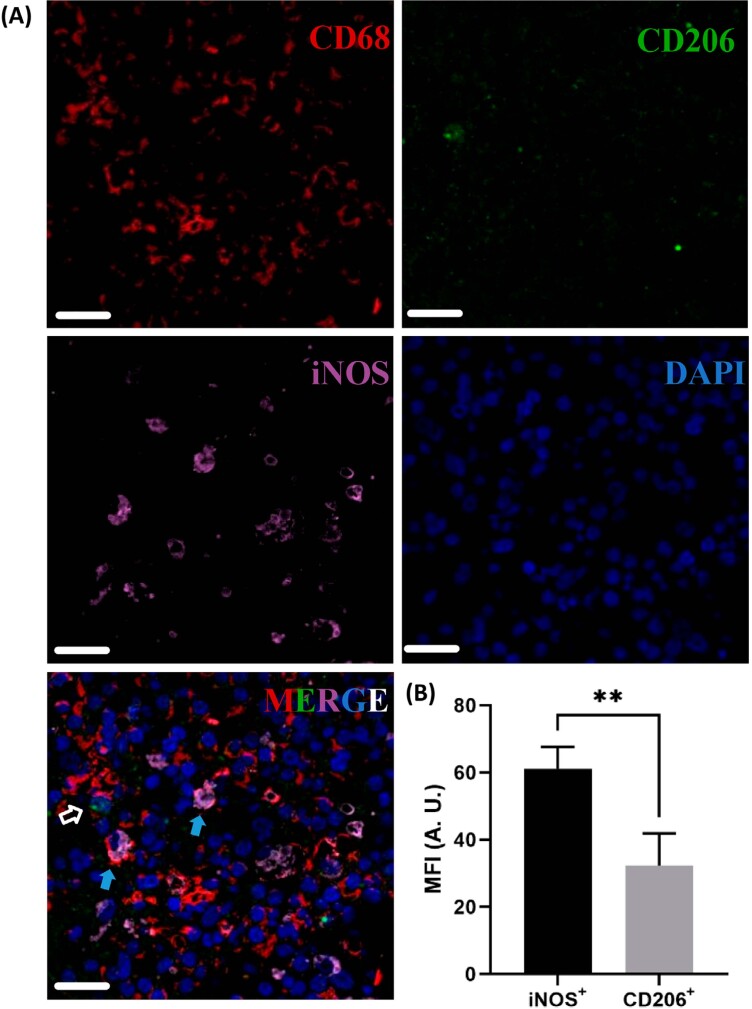
Polarized macrophage distributions in human apical periodontitis tissues. (A), representative TSA-based immunofluorescence staining images. (B), statistical results of the MFI analysis. CD68, general marker for macrophages; iNOS, specific marker for M1 macrophages; CD206, specific marker for M2 macrophages; DAPI, nuclear dye. In the merge picture, open white arrows indicate typical M2 macrophages (CD68+CD206+), blue arrows indicate typical M1 macrophages (CD68+iNOS+). MFI, mean fluorescence density; A.U., arbitrary unit. Scale bars, 20 μm. **p<0.01.

Representative TSA-based immunofluorescence staining images are shown in Figure 1A. As indicated by the CD68^+^ cells, macrophages were widely infiltrated in the human AP tissues. MFI analysis suggested that the M1 macrophages exhibiting the CD68^+^iNOS^+^ phenotype were significantly more abundant when compared with the M2 phenotype (CD68^+^CD206^+^) of macrophages (*p*<0.01) (Figure 1B) in human AP tissues.

### The IL-6/STAT3 pathway was highly activated in human AP tissues infected by *P. gingivalis*

**Figure 2:**
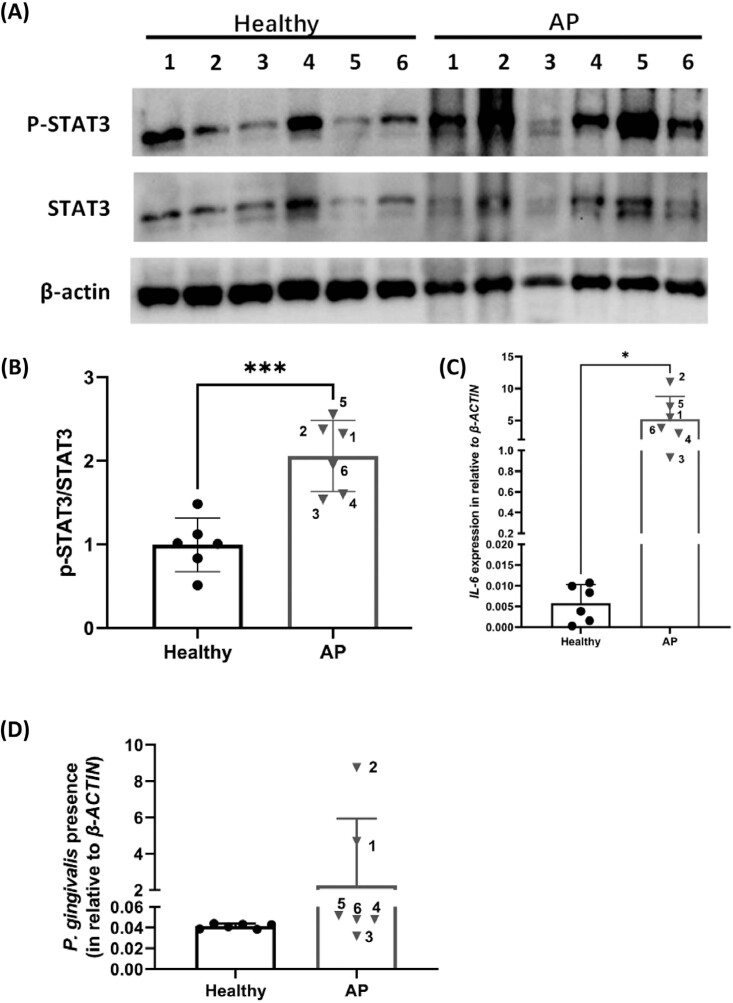
IL-6/STAT3 expression and *P. gingivalis* presence in human apical periodontitis tissues. (A), representative western blot images for p-STAT3, STAT3, and β-actin. (B), grayscale analysis of p-STAT3 expression (relative to STAT3). (C), gene expression level of IL-6 in healthy pulps and apical tissues. (D), *P. gingivalis* detection in apical tissues via RT-qPCR using universal *P. gingivalis* primers, showing that *P. gingivalis* were present in samples #1 and #2. Healthy, healthy human dental pulps; AP, apical periodontitis lesion tissues. Sample numbers are indicated beside the dots. *p<0.05; ***p<0.001

Western blotting of the human tissue samples indicated that phosphorylated STAT3 (p-STAT3) was highly expressed in AP tissues (*p*<0.001) (Figure 2A and B) when compared with the healthy tissues. RT-qPCR analysis revealed that the gene encoding IL-6 was also significantly induced in AP tissues (*p*<0.05) (Figure 2C), and the variation trend was in accordance with the one observed for p-STAT3 expression, as suggested by the correlation analysis (R=0.829, *p*<0.05) (data not shown). Considering that *P. gingivalis* is a major pathogen of AP^36^ and that *P. gingivalis* protein extracts can induce transcription of IL-6/STAT3 pathway-related genes in periodontal ligament cells,^37^ the presence of *P. gingivalis* in these AP tissues was investigated. Two (#1 & #2) of the six AP tissues tested positive for *P. gingivalis* infection via RT-qPCR of the extracted gDNAs, and these two samples also exhibited high-expression of p-STAT3 and IL-6 (Figure 2D), suggesting a potential relationship between *P. gingivalis* infection and activation of the IL-6/STAT3 pathway. However, the presence of *P. gingivalis* had no significant influence on the relative expression of macrophage M1 and M2 markers, as shown by the TSA staining results (supplementary file 1), suggesting that there might be other causal agents promoting the M1 polarization of macrophages in the absence of *P. gingivalis*.

### *P. gingivalis* infection induced IL-6 expression, STAT3 phosphorylation, and macrophage M1 polarization

**Figure 3:**
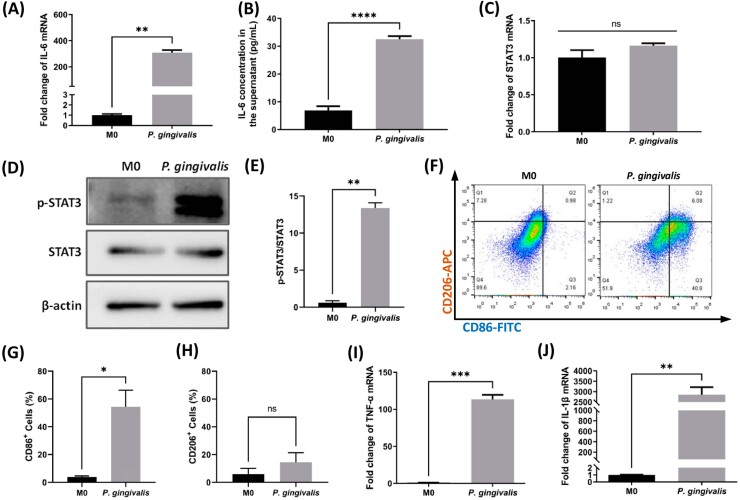
*P. gingivalis* infection induced IL-6 expression, STAT3 phosphorylation, and macrophage M1 polarization in BMDMs. (A-C) IL-6 transcription (A), IL-6 secretion (B), and Stat3 transcription (C) in response to *P. gingivalis* stimulation for 12 h. (D) and (E), p-STAT3 expression changes upon *P. gingivalis* activation. (F), representative flow cytometry results of unstimulated BMDMs (M0) and *P. gingivalis*-stimulated BMDMs. (G) and (H), statistical results for CD86+ (G) and CD206+ (H) macrophages in flow cytometry. (I) and (J), RT-qPCR results for expression levels of other M1 markers in response to *P. gingivalis* infection. M0, unstimulated BMDMs; *P. gingivalis*, BMDMs stimulated by *P. gingivalis* for 12 h. *p<0.05; **p<0.01; ***p<0.001; ****p<0.0001; ns, not significant

To confirm if *P. gingivalis* infection was effective in inducing IL-6/STAT3 pathway activation, as observed in the clinical samples, and how this reaction would manipulate macrophage polarization, *P. gingivalis* was co-cultured with BMDMs *in vitro*. RT-qPCR analysis demonstrated that, when compared with the unstimulated BMDMs, *P. gingivalis* infection of 12 h, at an MOI of 100, significantly upregulated the expression level of the gene encoding IL-6 by approximately 300-fold (*p*<0.01) (Figure 3A). This upregulation of IL-6 was confirmed by ELISA with a 4.6-fold average increase of IL-6 in the cell culture supernatant after 12 h of *P. gingivalis* infection (*p*<0.0001) (Figure 3B). Similar to the main signaling factor downstream of IL-6, the mRNA expression level of STAT3 remained stable upon stimulation with *P. gingivalis* (*p*>0.05) (Figure 3C). However, the expression of phosphorylated STAT3 increased significantly after 12 h of *P. gingivalis* infection (*p*<0.01) (Figure 3D and E), but decreased sharply after co-culturing with *P. gingivalis* for 24 h (data not shown). As p-STAT3 is the main effector of the IL-6/STAT3 pathway, 12 h – the point at which p-STAT3 expression peaked – was selected as the co-incubation period of *P. gingivalis* and BMDMs. The different trends of STAT3 mRNA and protein expression indicated that the phosphorylation status of STAT3, rather than the gene expression level of STAT3, was involved in the *P. gingivalis*-induced macrophage response.

IL-6 is one of the markers of the M1 macrophage phenotype.^17^ Therefore, polarization of BMDMs, with or without *P. gingivalis* engagement, was observed using FC. *P. gingivalis* stimulation for 12 h promoted 54.4±11.9% of BMDMs to differentiate towards the M1 phenotype (CD86^+^, as shown in quarters Q2 and Q3 of Figure 3F and G) (*p*<0.05), whereas the M2 type of differentiation was not significantly induced (CD206^+^, as evidenced in Q1 and Q2 of Figure 3F and H) (*p*>0.05). Other M1 differentiation markers, including IL-1β and TNF-α, were also significantly induced (*p*<0.01) upon *P. gingivalis* stimulation as indicated by RT-qPCR results (Figure 3 I and J).

### Phosphorylated STAT3 mediated M1 polarization of macrophages in the *P. gingivalis*-induced inflammation environment

Based on the finding that *P. gingivalis* infection induced STAT3 phosphorylation, rather than STAT3 transcription in macrophages, Stattic was added to investigate the role of the IL-6/STAT3 signaling pathway in the *P. gingivalis*-mediated macrophage polarization. Stattic is a nonpeptidic small molecule that selectively inhibits tyrosine 705 phosphorylation in STAT3.^38^ A working concentration of 5 μM Stattic was sufficient to inhibit phosphorylation of STAT3 while having a negligible influence on the growth and survival of BMDMs, as determined by Cell Counting Kit-8 (CCK8) test in our preliminary study (Supplementary file 2).

**Figure 4:**
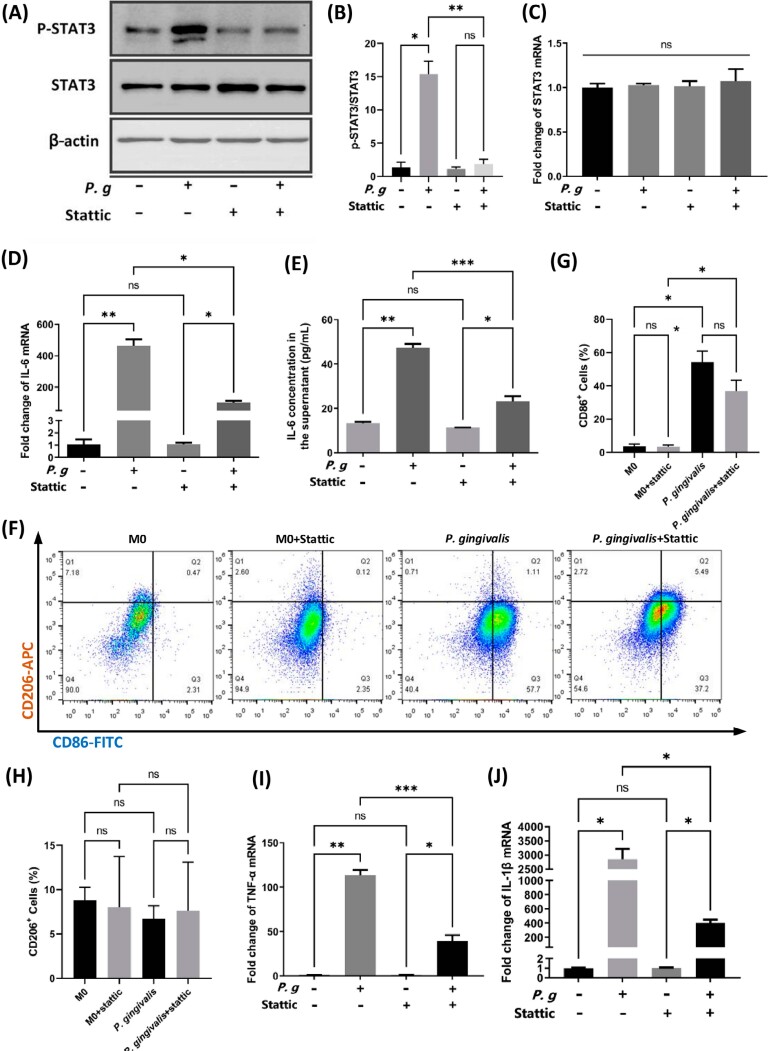
STAT3 blocking attenuated *P. gingivalis*-induced IL-6 production, STAT3 phosphorylation, and M1 type polarization in murine primary macrophages. (A), representative image demonstrating the phosphorylation status of STAT3 induced by *P. gingivalis* and suppressed by Stattic. (B), grayscale analysis of p-STAT3 expression (relative to STAT3). (C) and (D), relative mRNA expression levels of STAT3 (C) and IL-6 (D) after *P. gingivalis* infection for 12 h in the presence or absence of 5 μM Stattic. (E), levels of IL-6 protein secreted in the culture supernatant of BMDMs in response to *P. gingivalis* infection with or without 5 μM Stattic. (F), representative flow cytometry results exhibiting the influence of Stattic on macrophage polarization status after *P. gingivalis* stimulation for 12 h. (G) and (H), statistical results for CD86+ (G) and CD206+ (H) macrophages in flow cytometry. M0, unstimulated BMDMs; P. g and *P. gingivalis*, BMDMs stimulated by *P. gingivalis* for 12 h. *p<0.05; **p<0.01; ***p<0.001; ns, not significant

Stattic stimulation significantly inhibited the phosphorylation of STAT3 induced by *P. gingivalis* infection for 12 h (*p*<0.01) (Figure 4A and B) but had limited impact on STAT3 mRNA expression (*p*>0.05) (Figure 4C). *P. gingivalis*-derived induction of IL-6 mRNA transcription (Figure 4D) and protein secretion (Figure 4E) were also significantly suppressed (*p*<0.05) in the presence of 5 μM Stattic at the observed time point. This might be due to IL-6 production in macrophages also being mediated by the activated p-STAT3 feedforward autocrine feedback loop.^39^ Further investigations were conducted to ascertain if STAT3 blockade would reverse the macrophage phenotype switch under the influence of *P. gingivalis*. STAT3 blockade caused a 15% average reduction in the percentage of M1 (CD86^+^) macrophages after *P. gingivalis* infection for 12 h (*p*<0.05) in FC analysis (Figure 4F and G) but had little impact on the percentage of CD206^+^ macrophages (*p*>0.05) and the phenotypes of unstimulated BMDMs (*p*>0.05) (Figure 4F and H). Transcriptional induction of other M1 differentiation markers were also downregulated in the presence of Stattic (*p*<0.01) (Figure 4I and J). These findings suggested that *P. gingivalis* infection directed macrophage phenotype skewing, at least in part, through the IL-6/STAT3 pathway.

### Systemic STAT3 blockade alleviated apical bone resorption induced by *P. gingivalis* infection *in vivo*

**Figure 5:**
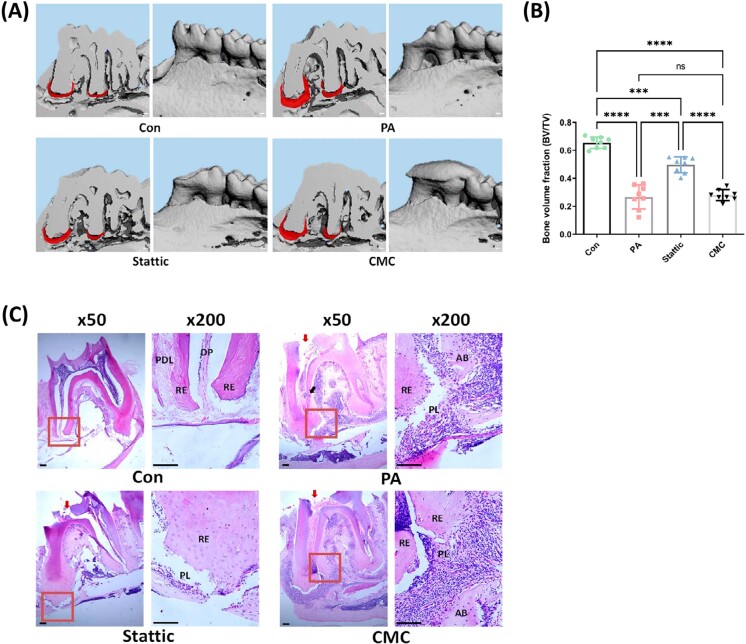
*P. gingivalis* infection caused radiographically detectable apical lesions *in vivo*, and systemic blockade of STAT3 alleviated bone resorption in *P. gingivali*s-derived murine apical periodontitis. (A), representative 3-D reconstruction images of the infected mandibles. Areas in red indicate the soft tissues surrounding the root apexes; scale bars, 100 μm. (B), analysis of the bone volume fractions (BV/TV). (C), representative H&E staining results for the apical regions. Scale bars, 100 μm. Con, control group; PA, *P. gingivalis*-induced apical periodontitis group; Stattic, Stattic (supplemented with 0.5% carboxymethylcellulose) treatment of the AP group; CMC, 0.5% carboxymethylcellulose treatment of the AP group. BV, bone volume; TV, tissue volume. ***p<0.001, ****p<0.0001; ns, not significant. Red arrows in the H&E staining pictures indicate pulpal accesses; black arrow in the AP group indicates lateral root canal. PDL, periodontal ligament; DP, dental pulp; RE, root end; AB, alveolar bone; PL, periapical lesion

As Stattic could reduce *P. gingivalis*-triggered expression of IL-6, which is an important cytokine in lytic bone diseases, the effect of STAT3 blockade on apical bone resorption was further explored using a *P. gingivalis*-derived murine AP model. Physical conditions of the mice remained stable in the 3 weeks of the experimental process as there were no significant differences in the body weight of mice in each group at the observed time points (0, 7, 14, 21 days after model construction) (Supplementary file 3). The 3-D reconstruction of the mandibles (Figure 5A) and calculation of the ratio of bone volume over tissue volume (BV/TV) (Figure 5B) suggested that periapical lesions were successfully induced in the right mandible of the mice by using the current method with *P. gingivalis* as the mono-species pathogen. The dimensions of radiolucency surrounding the mesial and distal root of the first molars were significantly larger in the AP group (0.1345±0.0193 mm^3^) when compared with the Con group (0.044±0.0028 mm^3^) (*p*<0.0001), and the average volume of bone was also significantly lower in the AP group, as implied by the BV/TV analysis. Oral gavage of Stattic prevented apical bone loss to some extent (*p*<0.001 for the comparison between the Stattic group and the AP group), with apical lesion dimensions for the Stattic group in the range of 0.0768±0.0051 mm^3^. BV/TV analysis also confirmed the protective effect of Stattic on the bone, whereas intragastrical administration of 0.5% CMC vehicle did not affect progression of apical lesions since the results for the CMC group (0.1528±0.0149 mm^3^) were similar to those of the AP group (*p*>0.05). Histological analysis (Figure 5C) indicated that the pulpal access was successfully generated without damaging the floor of the pulp chamber and also showed that the soft tissue surrounding the apical third of the teeth of the AP and CMC groups were more abundant when compared with those of the Con and Stattic groups, taken together with the CBCT scanning results, suggesting that apical bone destruction was more evident in the AP and CMC groups. Cells infiltrated in the periapical lesion (PL) site of the jawbone of the AP and CMC groups were more significantly stained blue instead of pink, suggesting a higher nuclear to cytoplasm ratio of the infiltrating cells, implicating the presence of inflammation.

### Stattic suppressed *P. gingivalis*-induced macrophage M1 polarization in the murine apical region

**Figure 6:**
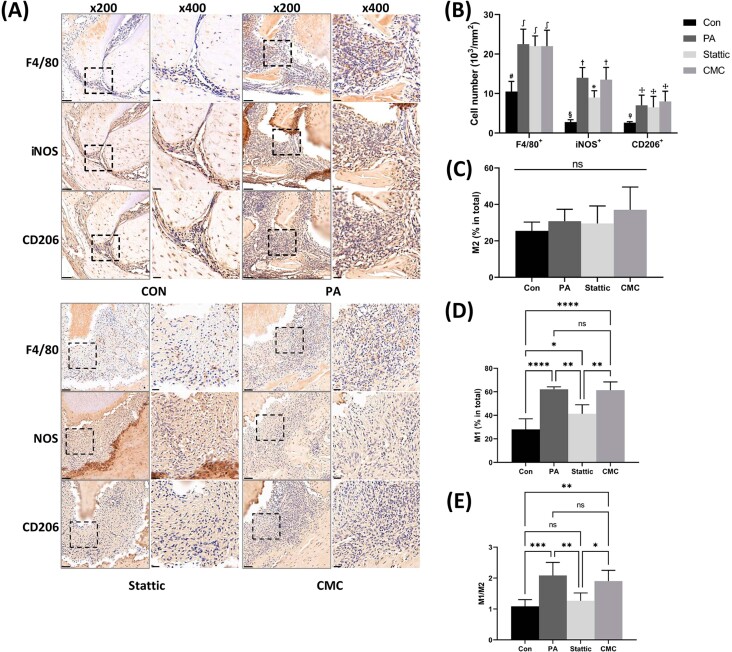
IHC staining of the apical regions of the mandibular first molars of the murine models. (A), representative IHC staining images of the Con, PA, Stattic, and CMC groups. Scale bars, 50 μm (×200) and 20 μm (×400), respectively. (B), statistical results for positive cell counting of the F4/80+, iNOS+, and CD206+ macrophages. Different markers on the bars indicate statistical differences. (C) and (D), percentages of the CD206+ (C) and iNOS+ (D) cells (relative to F4/80+ cells). (E) ratios of iNOS+ cells to CD206+ cells (M1/M2 ratios). Con, control group; PA, *P. gingivalis*-induced apical periodontitis group; Stattic, Stattic (supplemented with 0.5% carboxymethylcellulose) treatment of the AP group; CMC, 0.5% carboxymethylcellulose treatment of the AP group. *p<0.05; **p<0.01; ***p<0.001; ****p<0.0001; ns, not significant

Since the M1/M2 ratio is important in maintaining bone homeostasis, IHC staining was performed to assess the involvement of macrophages and their polarization status in *P. gingivalis*-induced bone destruction (Figure 6A). Positive cell counts of F4/80^+^ cells demonstrated that there was significantly more macrophage infiltration in the periapical area of the mandibular first molar in the AP, CMC, and Stattic groups when compared with the Con group (*p*<0.05) (Figure 6B). Counts of CD206^+^ cells, which represent M2 macrophages, were not statistically different among the PA, CMC, and Stattic groups (*p*>0.05) (Figures 6B and C). However, cells that stained positive for iNOS, which labeled the M1 macrophages, were significantly more abundant in the AP and CMC groups when compared with the Stattic group (*p*<0.01), suggesting a greater presence of M1 macrophages in the former two groups (Figures 6B and D). The AP and CMC groups exhibited an increasing trend in M1/M2 ratio relative to the Con and Stattic groups (*p*<0.05) (Figure 6E). These observations suggested that *P. gingivalis-*induced AP enhanced macrophage infiltration in the apical region of the affected teeth, and slightly upregulated the M1/M2 ratio in the lesion site. Furthermore, Stattic restored the apical M1/M2 ratio through suppressing M1 polarization of macrophages in response to *P. gingivalis* infection, which may contribute to the bone preservation effect of Stattic.

### *P. gingivalis*-infected macrophages have the potential to disturb bone metabolism

**Figure 7:**
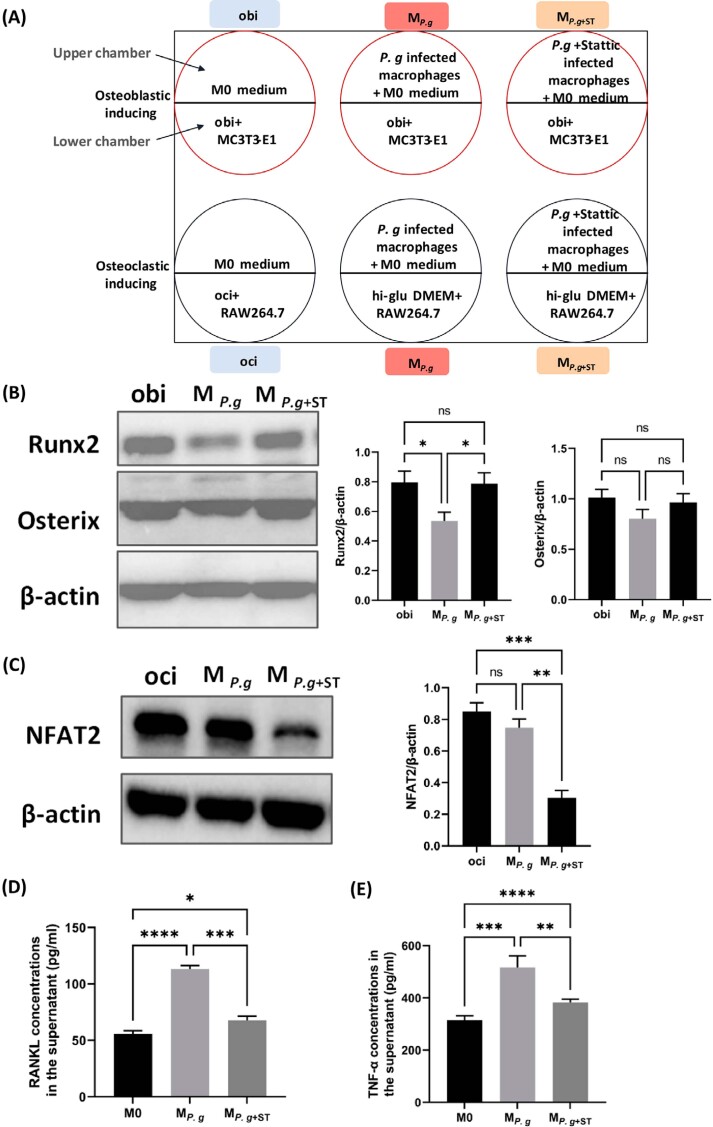
Effects of *P. gingivalis*-infected macrophages on protein expression levels in MC3T3-E1 and RAW264.7. (A), schematic diagram of the co-culture system. (B), representative bands and grayscale analysis for Runx2 and Osterix protein expression levels in MC3T3-E1 cells. (C), representative bands and grayscale analysis for NFAT2 protein expression levels in RAW264.7 cells. (D) and (E), TNF-α (D) and RANKL (E) production in BMDMs after *P. gingivalis* infection for 12 h with or without 5 μM Stattic. M0, unstimulated BMDMs; MP.g, BMDMs stimulated with *P. gingivalis* for 12 h; MP.g+ST, BMDMs stimulated with *P. gingivalis* in the presence of 5 μM Stattic for 12 h. obi, osteoblastic-induced medium; hi-glu DMEM, complete high-glucose DMEM medium; oci, osteoclastic-induced medium, composed of hi-glu DMEM supplemented with 50 ng mL-1 RANKL. *p<0.05; **p<0.01; ***p<0.001; ****p<0.0001; ns, not significant

To further characterize the role of macrophages in apical bone destruction caused by *P. gingivalis* infection, the effects of *P. gingivalis*-infected macrophages on both osteoblastic- and osteoclastic-inducing processes were investigated via a Transwell co-culture system (Figure 7A). A *P. gingivalis* survival experiment performed in our preliminary study demonstrated that at a MOI of 1:100, *P. gingivalis* could invade and survive inside BMDMs for up to 3 days (Supplementary file 4). Thus, 3 days was selected as the period for co-culturing via Transwells. Western blotting showed that, after three days of co-culture with *P. gingivalis-*infected BMDMs, cells of the pre-osteoblast cell line MC3T3-E1 exhibited decreased expression of the early phase osteogenic-related protein Runx2 (*p*<0.05), but had little influence on the expression of Osterix (*p*>0.05) (Figure 7B, bands of the M_P.g_ group). Furthermore, the inhibition effects were abrogated when BMDMs were infected with *P. gingivalis* in the presence of 5 μM Stattic (*p*<0.05) (Figure 7B, bands of the M_P.g+ST_ group). RAW264.7 cells co-cultured with *P. gingivalis-*infected BMDMs expressed a comparable level of nuclear factor of activated T cells 2 (NFAT2) (Figure 7C, bands of the M_P.g_ group), the master regulator of osteoclastogenesis, to the RANKL-induced positive controls (*p*>0.05) (Figure 7C, bands of the oci group). However, BMDMs treated with *P. gingivalis* plus Stattic lost the ability to trigger NFAT2 expression in RAW264.7 cells (*p*<0.01) (Figure 7C, bands of the M_P.g+ST_ group). These findings suggested that *P. gingivalis*-infected BMDMs had the potential to disrupt the bone metabolism process indirectly. To dissect which component was conducive to these disturbances, the concentrations of TNF-α and RANKL, two major factors involved in osteoclast generation,^40,41^ were measured in the supernatants of *P. gingivalis*-stimulated BMDMs using ELISA. After 12 h of *P. gingivalis* infection, BMDMs produced significantly more RANKL and TNF-α compared with the unstimulated M0 group (*p*<0.001), while addition of Stattic effectively dampened the induction of RANKL and TNF-α secretion (*p*<0.01). These results indicated the potential involvement of RANKL and TNF-α, derived from *P. gingivalis* stimulation, in suppressing osteogenesis and provoking osteoclastogenesis (Figures 7D and E).

## Discussion

**Figure 8:**
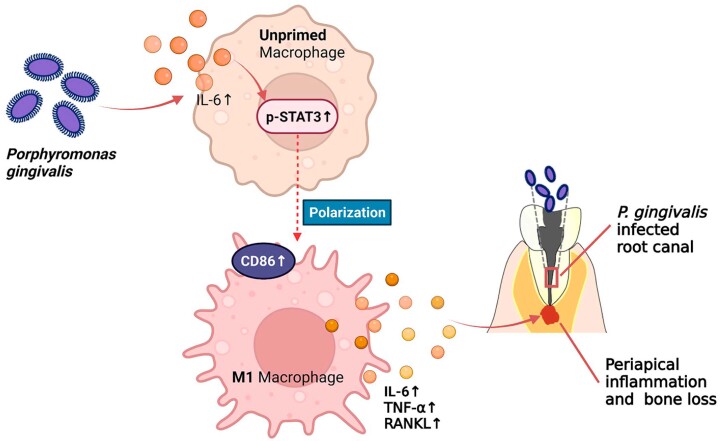
Schematic illustration. *P. gingivalis* infection polarizes macrophages toward the M1 phenotype via activating the IL-6/STAT3 pathway. The M1 macrophages accelerate apical inflammation and bone destruction via secretion of IL-6, TNF-α, and RANKL, which are pro-inflammatory mediators that can impede osteogenesis and promote osteoclastogenesis

This study investigated the relationship between the IL-6/STAT3 signaling pathway and macrophage polarization in a *P. gingivalis*-stimulated apical inflammatory microenvironment. Findings from the study suggest that *P. gingivalis* may induce apical bone resorption via recruitment and enhancing the M1 polarization of macrophages, a process that is mediated by the IL-6/STAT3 signaling pathway (Figure 8).

Macrophages share a common myeloid origin and microenvironment with osteoclasts,^42^ and are also prominent players in maintaining bone homeostasis.^43,44^ An increased M1/M2 ratio is related to enhanced osteoclastic activity and production of pro-inflammatory mediators.^19,45^ As indispensable infiltrates in apical periodontitis, an oral lytic bone disease, the distributions and quantities of M1 and M2 macrophages in AP tissues have been explored in several studies. However, these studies either compared the M1 proportions in radicular cysts and in apical granuloma,^4^ which lack direct comparison between the M1 and M2 subpopulations in the same pathological state; or appraised the M1/M2 ratio via only a general marker of macrophages (CD68) and an M2-specific marker (CD163),^23^ which may cause bias when quantifying the M1 percentage. In our study, both CD68^+^iNOS^+^ M1 and CD68^+^CD206^+^ M2 subtypes of macrophages were quantified in periapical lesion tissues collected from patients with pre-treated AP plus intact coronal restoration and no signs of root fracture, which suggested persistent and uncontrolled infection.^46,47^ Infiltrated M1 macrophages in these apical lesion tissues were significantly more abundant compared with M2 macrophages. IHC staining of the apical tissues surrounding the mandibular first molar of the murine AP models also exhibited an increasing trend in the M1/M2 ratio after AP induction. These results were congruent with observations reported by Weber, et al.^24^ (2018). Furthermore, through evaluation of the tissue expression of IL-4, IL-12, and IFN-γ, Fraga and colleagues found that the IFN-γ protein expression was increased in radicular cysts.^48^ IFN-γ belongs to the family of T helper (Th) 1-like cytokines and is intimately connected with the M1 polarization of macrophages.^17^ These findings implied the active participation of M1 macrophages in tissue damage and inflammatory progress.

The ability of the IL-6/STAT3 pathway to induce M1 polarization of macrophages contradicted previous reports.^49,50^ IL-6, as an important pro-inflammatory cytokine, is also a major player in chronic inflammatory diseases, autoimmune diseases, cancer, and cytokine storm.^25^ STAT3 is the principal signaling factor downstream of IL-6.^35^ Via phosphorylation of Tyr705, STAT3 is translocated into the nucleus where the activated STAT3 can bind to the promoter regions of numerous target genes to induce the transcription of diverse regulators that are involved in cellular proliferation, cell survival, microvascular proliferation, and the production of IL-6.^39,51^ The ability of STAT3 to promote *IL-6* gene expression was concluded as a feedforward autocrine/paracrine feedback loop.^52^ IL-6 was previously concluded to mediate M2 polarization of macrophages, but these studies were either conducted based on stimulations derived from cancer cells,^49,53^ or through adding exogenous IL-6 to induce the phenotype shift in macrophages.^54^ *P. gingivalis* is known to robustly enhance the expression of IL-6 in macrophages,^11,18^ and protein extracts of *P. gingivalis* up-regulated the transcription of 29 genes belonging to the IL-6/STAT3 pathway, including *IL-6* ^37^, which was consistent with our results. In our study, activation of STAT3 and the M1 type skewing were strongly enhanced upon *P. gingivalis* stimulation, while IL-6 induction and M1 polarization were attenuated in the presence of the STAT3 blocker, Stattic. These *ex-vivo* results suggested the possible involvement of the IL-6/STAT3 signaling pathway in *P. gingivalis*-mediated M1 polarization of macrophages, which contradicts previously reported observations. These discrepancies might be due to the inherent complexity of the hyperinflammatory stress conferred by *P. gingivalis*, a versatile pathogen that has the capacity to interact with various macrophage cell surface receptors, including TLRs, and protease-activated receptors (PARs), leading to multifunctional and/or phenotypic changes of macrophages.^18^ The autocrine origin of the IL-6 in our experiment might also be a potential motivator that drives macrophages toward the M1, rather than the M2, phenotype.

The murine AP model in the current study showed that the M1 phenotype of macrophages was associated with more significant bone lesions. The M1 subtype of macrophages secrete high levels of pro-inflammatory cytokines including IL-6, TNF-α, IL-1β, and granulocyte-macrophage colony-stimulating factor (GM-CSF), which accelerate osteoclastic differentiation and bone destruction via induction of RANKL expression.^43^ Highly activated M1 macrophages are implicated in the progression of bone-resorbing diseases such as rheumatoid arthritis, osteoporosis, and osteonecrosis of the jaw.^45,55,56^ In our study, the *P. gingivalis*-stimulated macrophages exhibited an M1 phenotype and expressed high levels of IL-6, TNF-α, and RANKL as measured by ELISA. TNF-α and IL-6 are both positive regulators of osteoclastogenesis and negative regulators of osteoblastogenesis.^42^ IL-6 can promote RANKL production in osteoblasts via activating STAT3.^42^ TNF-α can also induce RANKL and IL-1α secretion in osteoblasts and marrow stromal cells.^13^ Furthermore, TNF-α can work synergistically with or without RANKL to trigger expression of NFAT2 (master mediator of osteoclastogenesis) and the subsequent osteoclast differentiation in osteoclast precursor cells.^42,57,58^ Moreover, in the presence of pre-osteoblasts, TNF-α downregulated the expression of Runx2 in the early phase of osteoblast differentiation.^42,57^ Fahy, et al.^59^ (2014) reported that conditioned medium of M1 polarized macrophages significantly inhibited the expression of genes involved in chondrogenesis in mesenchymal stem cells. This was consistent with observations from the Transwell co-culture system in this study, indicating that the *P. gingivalis*-infected macrophages may adopt pro-inflammatory mediators to interfere with bone metabolism and promote bone destruction.

As *P. gingivalis* infection activated STAT3 phosphorylation in macrophages, rather than STAT3 gene transcription, Stattic was utilized to analyze the role of the IL-6/STAT3 pathway in macrophage responses derived from *P. gingivalis* stimulation. Stattic was selected because it is a selective STAT3 tyrosine phosphorylation inhibitor^38^ that has little impact on the phosphorylation states of other molecules in the STAT family and relevant members of the JAK, Akt, and ERK pathways.^60,61^ Stattic partially blocked the induction of IL-6 in macrophages *in vitro* in the current study, implying the involvement of the STAT3 signaling pathway in *P. gingivalis*-mediated inflammation. The inhibition of STAT3 activation further alleviated apical bone resorption as shown in the experimental murine model of AP. The safety of Stattic administration has been proved by numerous studies.^35,62,63^ Similarly to our study, Latourte et al.^35^ (2017) reported bone protection effects of Stattic in experimental osteoarthritis, a disease that also frequently manifests enhanced bone resorption. Li, et al.^29^ (2018) demonstrated that Stattic exhibited anti-osteoclastogenesis properties and inhibited bone resorption in RANKL-induced RAW264.7 cells in a dose-dependent manner. These findings, congruent with results from the current study that show the suppression effects of Stattic on macrophage production of osteoclastogenesis mediators, highlight the intimate involvement of the activated IL-6/STAT3 signaling pathway in bone-destructive diseases, and further contribute to improved understanding of the physiology and pathogenesis of AP.

*P. gingivalis*, as one of the most frequently detected pathogens in infected root canals, failed to cause periodontitis in germ-free mice,^64^ thus raising questions about whether *P. gingivalis* mono-species infection would cause AP severe enough for radiographic detection. By using the method described herein, a model of AP derived from *P. gingivalis* infection was successfully established. During pulp chamber access, part of the coronal pulp was destroyed so that the inoculated *P. gingivalis* could directly contact blood and decomposed tissues. Additionally, the pulp chamber served as a natural biological niche for *P. gingivalis* colonization, thereby facilitating the propagation and pathogenesis of *P. gingivalis*. When compared with the widely used “left open” models,^65–67^ which directly expose the pulp chamber to the oral environment to induce multi-species mixed endodontic infection, the model constructed for our study enabled more accurate analysis of the precise effects that the versatile pathogen *P. gingivalis* exerted on the progression and manifestation of AP.

## Conclusions

The presence of M1 macrophages and IL-6/STAT3 expression was increased in lesion tissues of patients with chronic AP. *P. gingivalis* stimulation robustly induced IL-6 expression and STAT3 activation in BMDMs, and activation of the IL-6/STAT3 pathway mediated *P. gingivalis*-derived M1 polarization of BMDMs. *P. gingivalis* mono-species infection could cause detectable AP, recruiting macrophages to the apical region and directing their M1 polarization. The *P. gingivalis*-infected BMDMs may accelerate bone destruction via secretion of IL-6, TNF-α, and RANKL, which hinder osteogenesis and promote osteoclastogenesis. The IL-6/STAT3 pathway is involved in mediating M1 polarization in the apical region and production of pro-inflammatory cytokines from *P. gingivalis*-infected BMDMs.
